# Impact of Collagen Peptide Supplementation in Combination with Long-Term Physical Training on Strength, Musculotendinous Remodeling, Functional Recovery, and Body Composition in Healthy Adults: A Systematic Review with Meta-analysis

**DOI:** 10.1007/s40279-024-02079-0

**Published:** 2024-07-26

**Authors:** Kevin Bischof, Anna Maria Moitzi, Savvas Stafilidis, Daniel König

**Affiliations:** 1 Section for Nutrition, Exercise and Health, Department for Nutrition, Faculty of Life Sciences, University of Vienna, Vienna, Austria; 2 Section for Nutrition, Exercise and Health, Department of Sports Science, Centre for Sports Science and University Sports, University of Vienna, Vienna, Austria; 3 Department for Biomechanics, Kinesiology and Computer Science in Sport, Centre for Sports Science and University Sports, University of Vienna, Vienna, Austria; 4 Vienna Doctoral School of Pharmaceutical, Nutritional and Sport Sciences, University of Vienna, Vienna, Austria

## Abstract

**Introduction:**

Over the past decade, collagen peptide (CP) supplements have received considerable attention in sports nutrition research. These supplements have shown promising results in improving personal health, enhancing athletic performance, and preventing injuries in some but not all studies.

**Objective:**

A systematic review and meta-analysis of randomized controlled trials (RCTs) has been conducted to investigate the effects of long-term daily collagen peptide (CP) supplementation on strength, musculotendinous adaptation, functional recovery, and body composition in healthy adults, both with and without concurrent exercise interventions over several weeks.

**Methods:**

The PRISMA with PERSiST guidelines were followed for this systematic literature review, which was conducted in December 2023 using PubMed, Scopus, CINAHL, and SPORTDiscus databases. Eligible studies included healthy, normal to overweight adults over 17 years of age who engaged in exercise and daily collagen peptide (CP) supplementation for a minimum of 8 weeks (except one 3-week trial only included for maximal strength). Studies examining recovery-related outcomes were also eligible if they included a 1-week supplementation period without exercise. Methodological study quality was assessed using the PEDro scale. A random-effects model with standardized mean differences (SMD) of change scores was chosen to calculate overall effect sizes.

**Results:**

Nineteen studies comprising 768 participants were included in both the systematic review and meta-analysis. Results indicate statistically significant effects in favor of long-term CP intake regarding fat-free mass (FFM) (SMD 0.48, *p* < 0.01), tendon morphology (SMD 0.67, *p* < 0.01), muscle architecture (SMD 0.39, *p* < 0.01), maximal strength (SMD 0.19, *p* < 0.01), and 48 h recovery in reactive strength following exercise-induced muscle damage (SMD 0.43, *p* = 0.045). The GRADE approach revealed a moderate certainty of evidence for body composition, a very low certainty for tendon morphology and mechanical properties, and a low certainty for the remaining.

**Conclusion:**

This systematic review and meta-analysis represents the first comprehensive investigation into the effects of long-term CP supplementation combined with regular physical training on various aspects of musculoskeletal health in adults. The findings indicate significant, though of low to moderate certainty, evidence of improvements in fat-free mass (FFM), tendon morphology, muscle mass, maximal strength, and recovery in reactive strength following exercise-induced muscle damage. However, further research is required to fully understand the mechanisms underlying these effects, particularly regarding tendon mechanical properties and short-term adaptations to collagen peptide (CP) intake without exercise, as observed in recovery outcomes. Overall, CP supplementation appears promising as a beneficial adjunct to physical training for enhancing musculoskeletal performance in adults.

Open Science Framework (Registration DOI: https://doi.org/10.17605/OSF.IO/WCF4Y).

**Supplementary Information:**

The online version contains supplementary material available at 10.1007/s40279-024-02079-0.

## Key Points

**Table:** 

Long-term intake of collagen peptide supplementation in conjunction with resistance or concurrent training seems to offer advantages for active individuals and athletes aiming to improve fat-free mass, maximal strength, tendon morphology, and reactive strength recovery.
Enhancing the tendinous cross-sectional area through collagen peptides has the potential to act preventively against sports-related tendon injuries.
Based on current data, achieving the desired adaptations seems to require a daily intake of 15 g of CP for at least 8 weeks; however, more research is needed on this topic.

## Introduction

Recently, global sales of food supplements, in particular of collagen peptides (CP), have surged tremendously, reaching a market value of 599 million USD in 2020 [1]. This trend underscores an urgent need for comprehensive scientific research to evaluate the current evidence on (CP) usage, particularly in the domains of sports nutrition and rehabilitation. The need for systematic scientific assessment is further emphasized by the increasing number of publications on this topic.

At present, 40 mammalian collagen genes have been identified, encoding 29 specific collagen types, with types I and III being the most abundant in muscles [2, 3]. Myofibrillar proteins are crucial for generating force, while collagenous tissue, such as intramuscular connective tissue, plays a significant role in force transmission, underscoring its importance in overall force output [4]. Given the repetitive amino acid sequence (Gly–X–Y) and the resulting abundance of glycine, proline (Pro), and hydroxyproline (Hyp) in collagen proteins, the current rationale for potential effects of CP supplementation may hinge on the integration of these amino acids into musculotendinous tissues derived from the ingested CP [5, 6]. Shaw and colleagues [7] have already reported a significant uptake of these particular amino acids within 2 h after administration of 15 g gelatin and 6 min of rope-skipping compared with placebo in human serum. Furthermore, both collagen content and concentration increased significantly in engineered human ligaments after the ingestion of 15 g [7]. Postprandial absorption trials without exercise demonstrated peak concentrations of certain collagen di- and tripeptides in human blood [8] and mouse skin [9] after 60 and 15 min, respectively. Furthermore, it has been shown that absorption of enzymatically hydrolyzed collagen protein is higher than that of nonhydrolyzed collagen [10]. Prolonged daily CP supplementation resulted in higher plasma Hyp peptide levels 1 and 2 weeks following the initial CP intake in young and older adults (20–60 years) [11]. Mouse skin fibroblasts [12] and cartilage tissue [13] also exhibited elevated levels of Pro–Hyp and Pro, respectively, after acute collagen administration, indicating an overall enhanced uptake of specific CPs. Notably, as gelatin and CPs are sometimes used interchangeably, water-soluble gelatin is derived from heat-exposed and hydrolyzed collagen [14], which can be further processed into collagen peptides through enzymatic breakdown [15]. Compared with gelatin (~ 100 kDa) [16], CPs generally have a lower molecular weight (~ 0.5–3 kDa) [17], exhibit serum peptidase resistance, and have been observed to be absorbed in larger portions than tripeptides [18]. This enhances the tissue incorporation of consumed CPs and potentially elicits biological effects as bioactive peptides.

Protein supplementation in combination with resistance training (RT) is known to increase lean body mass and reduce fat mass as demonstrated by a recent meta-analysis that primarily included studies utilizing protein sources rich in essential amino acids (EAA) (e.g., whey protein) [19]. However, CPs are generally considered low in EAAs and are often categorized as low-quality protein [20]. Despite this, CP supplementation has shown significant impacts on fat-free mass (FFM) in various populations. For example, in older adults, daily ingestion of 40 g of CP, whey protein, or whey protein combined with RT over the course of a year did not result in differences in FFM [21]. A 12-week RT program with middle-aged, untrained men supplementing 15 g of CP daily led to a significant increase in FFM and loss in fat mass (FM) compared with a placebo [22]. Similarly, premenopausal women participating in a 12-week concurrent training (CT) regimen experienced significant gains in FFM when supplemented with 15 g of CP [23]. Additionally, a significant loss of FM and augmentation in FFM has been reported in elderly sarcopenic men [24]. A combination of (predominantly) resistance training (RT) and timely CP supplementation appears to provide the strongest stimulus for upregulating anabolic pathways such as phosphatidylinositol 3-kinase–protein kinase B (PI3k-Akt), mitogen-activated protein kinase (MAPK), and the mechanistic target of rapamycin (mTOR) to induce myofibrillar hypertrophy and collagen synthesis [25]. Some trials also included vitamin C supplementation alongside CPs, as there is limited evidence demonstrating increased collagen type I synthesis and reduced oxidative stress [26, 27].

Numerous exercise intervention studies have investigated the potential benefits of regular CP intake on musculotendinous adaptation processes; however, the results were not always consistent. For instance, a study involving 15 weeks of lower-body resistance training (RT) found a significantly greater extent of myofibrillar hypertrophy in the vastus medialis, as measured by magnetic resonance imaging, with daily intake of 15 g of CP compared with a placebo. However, this effect was not observed in the overall exercised muscles (quadriceps + hamstrings + gluteus maximus) [28]. In another study assessing both muscle cross-sectional area (CSA) and thickness, CP supplementation led to similar increases in vastus lateralis following a 10-week RT intervention compared with a mixture of whey protein and creatine [29]. Long-term experiments investigating tendinous remodeling have also demonstrated equivocal outcomes. For example, intake of 15 g of CP over more than 3 months, combined with RT, did not produce superior gains in patellar tendon CSA or stiffness [30]. In contrast, 14 weeks of 5 g of CP alongside a similar exercise intervention showed significantly greater CSA in both Achilles and patellar tendons [31, 32].

As tendons play a pivotal role in transferring contractile force, the influence of CP intake on biomechanical parameters such as maximal voluntary contraction (MVC) and reactive strength, usually determined by countermovement jump (CMJ) height, has also been investigated in healthy adults. A study employing a 12-week strength training protocol combined with daily administration of 15 g of CP demonstrated similar gains in leg strength (leg press) and significantly higher handgrip strength compared with a placebo [33]. The rate of force development (RFD) in collegiate athletes has also been significantly improved following 3 weeks of RT and 20 g of CP [34]. Moreover, in a study focused on functional recovery, the restoration of baseline CMJ height following exercise-induced muscle damage was significantly enhanced after 12 weeks of CT and 15 g CP [35]. Muscle soreness, serving as an indicator of muscle damage, has also been extensively investigated in the context of CP supplementation but yielded contradictory results [36–38] likely influenced by varying study designs, especially differences in the duration of supplemental interventions.

So far, just one systematic review without meta-analyses examined possible effects of CP supplementation on body composition, collagen synthesis, and recovery from joint injury and exercise [39]. The authors concluded that 15 g daily appeared to be effective for improving body composition as well as enhancing collagen synthesis in respective tissues; in addition, they found that CP intake might also moderately improve muscle recovery [39]. Since then, several studies focusing on CP supplementation in the field of sports nutrition have been published. The fact that study populations, administered dosages, outcome parameters, and durations contain at least some heterogeneity, a meta-analysis has been conducted, aiming for a comprehensive statistical investigation of the aforementioned aspects. Both comprehensive quantitative and qualitative research performed in this review aims to reveal whether CP intake linked with exercise, in particular RT, is associated with improvements in sports performance-related injury-preventive conditions. Therefore, we analyzed the extent of remodeling in body composition, muscles, and tendons and possible adaptation in strength and biomechanical recovery-related outcomes.

## Methods

The present systematic review and meta-analysis was prospectively registered at Open Science Framework (Registration DOI: https://doi.org/10.17605/OSF.IO/WCF4Y) and written on the basis of PERSiST (implementing Prisma in Exercise, Rehabilitation, Sport medicine and SporTs science) guidelines [40].

### Eligibility Criteria

Studies investigating healthy female and male adults (> 17 years of age) with a lower and upper body mass index (BMI) limit of 18.5–31 kg/m^2^ (a BMI of 31 due to one study being also suitable for this meta-analysis [22]) and no preceded periods of ankle or joint pain were included. Both low and professional training status (sedentary to five trainings a week) of subjects were suitable for inclusion. Participants had to ingest a daily dose of CP (as treatment group) for at least 3 weeks, and the control group received a calorie- or non-calorie-matched placebo regardless of manufacturer and added vitamin C. As one article appeared suitable with a supplementation regimen of three times a week, this one was also considered for meta-analysis [41]. All included studies exclusively administered CPs instead of gelatin. To be also eligible, no energy restriction (e.g., phases of hypoenergetic states) during the intervention period was allowed. Moreover, only studies with an accompanying training intervention, either endurance, resistance, or concurrent training, under normobaric conditions of at least two times a week and a minimum of 3 weeks were included (except recovery-related studies, where duration of the supplementation phase was constituted to a minimum of 1 week without obligatory training intervention). Manuscripts had to be English- or German-written randomized controlled trials (RCT). Animal, in vitro, and ex vivo studies were excluded. Studies adding creatine or caffeine as fortification were also excluded.

### Information Sources and Search Strategy

**Fig. 1:**
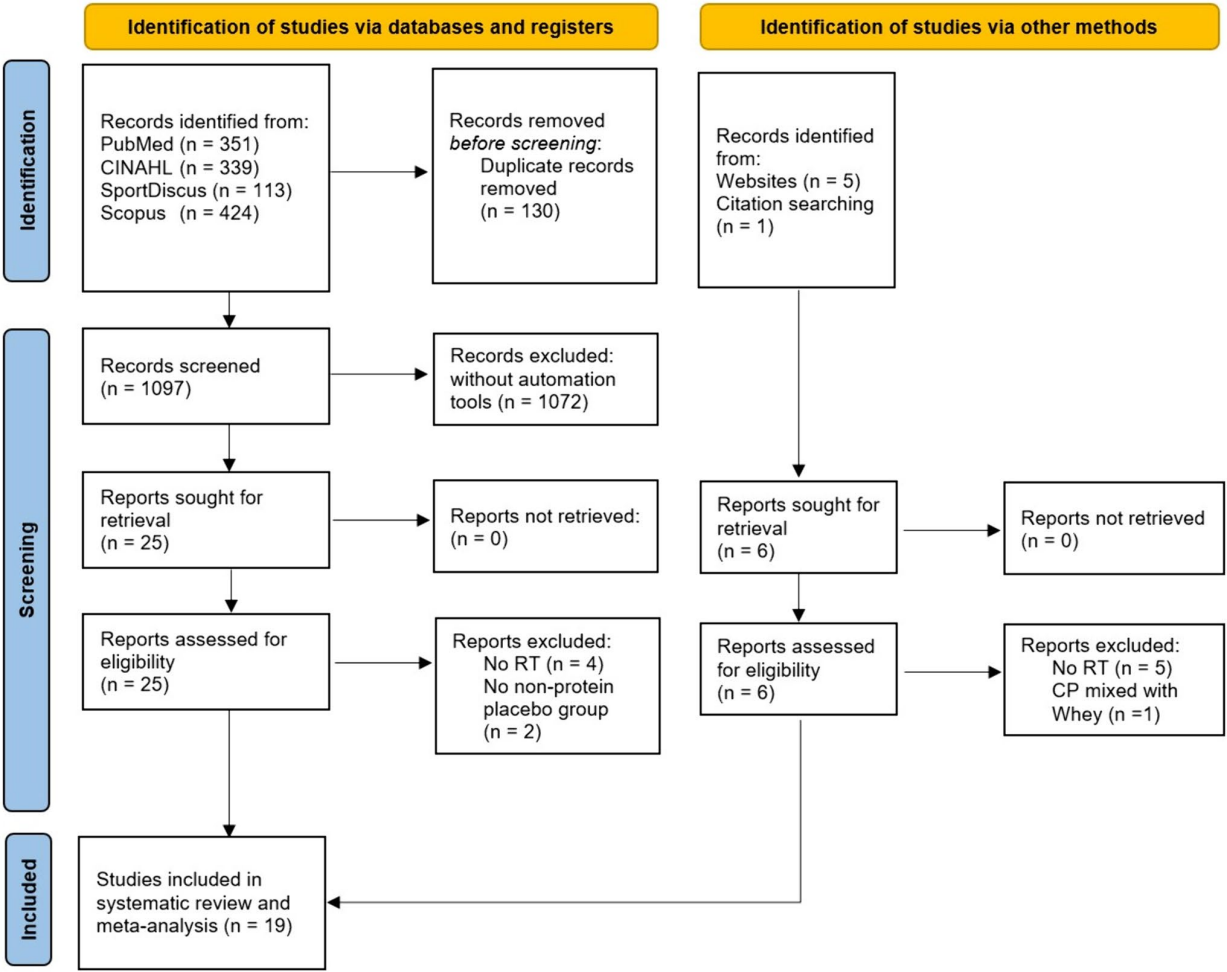
PRISMA flow chart

Literature search was carried out in December 2023 in PubMed, Scopus, CINAHL, and SPORTDiscus (last both via EBSCO) without restrictions regarding publication year, language, and authors—only any kind of review was excluded via filters. A search string was applied in all databases: “collagen AND (peptide OR peptides OR supplement OR supplementation OR hydrolysate) AND (recovery OR muscle strength OR body composition OR architecture OR tendon OR muscle OR performance) [AND (only added in Scopus)] NOT (disease OR skin OR osteoporosis OR osteoarthritis).” “Clinical Trials” and “Randomized Control Trials” filters in PubMed and “Article title, Abstract, Keywords” instead of “All fields” in Scopus were additionally used for further specification. References of included studies as well as Google Scholar were also screened (forward and backward search) and, in case of eligibility, handpicked. Gray literature was not actively sought but considered if eligibility criteria were met. The results of the search process are illustrated in Fig. 1.

### Selection and Data Collection Process

After initial search string query in all databases, literature screening was performed by two independent reviewers (A.M.M., K.B.) with the free online Rayyan tool (https://www.rayyan.ai/). If discrepancies of in-/exclusion of articles occurred following the screening of title, abstract and full-text, the two reviewers resolved them by consensus. If not, a third reviewer (S.S.) made the final decision. Data of included studies were collected by K.B. from articles directly or authors upon request (in most cases).

### Data Extraction

Number of participants, study design, age, training status, dose, type of training intervention, and placebo as well as outcome parameters were extracted from included studies and can be located in Table 1. If data could not be found inside articles, corresponding authors were contacted via email for request. For one particular study [38], WebPlotDigitizer (https://automeris.io/WebPlotDigitizer/) was used to extract mean ± standard deviation (SD) from graphs. If only standard error (SE) instead of SD could be found, SD was calculated by $\text{SD}=\text{SE}\times \sqrt{N}$ [42]. In regard to the meta-analysis, the following individual parameters per study were chosen for each item. Fat-free mass: whole body fat-free mass obtained via dual-energy X-ray absorptiometry (DXA) or bioelectrical impedance analysis (BIA). Muscular adaptation: volume (cm^3^) of quadriceps (and also rectus femoris, vastus intermedius, lateralis and medialis alone), gluteus maximus and hamstrings; CSA (cm^2^) of the thighs; thickness (cm) of medial gastrocnemius, vastus lateralis, intermedius and rectus femoris. Tendinous remodeling: (a) tendon functional properties: Young’s modulus (GPa) of patellar tendon (PT) and stiffness (N/mm) of PT and Achilles tendon (AT), (b) tendon morphological properties: CSA (mm^2^) of PT and AT. Body composition: fat-free mass (FFM) (kg). Strength: MVC (Nm, N) of knee extension and flexion and plantar flexion using a dynamometer or 90° leg press; one-repetition maximum (1RM) (kg) of squat, deadlift, bench press, bent-over row (all with barbells), knee extension on a dynamometer and handgrip with a hand dynamometer; maximal isometric squat performance (%) and squat jump (N), both assessed via force plates. Recovery: MVC (Nm) of knee extensions using a dynamometer, CMJ height (cm) with force plates or optical instruments, muscle soreness (MS) (mm) by means of a visual analog scale—values from post, 24 h and 48 h after exercise-induced muscle damage. Since only two studies investigated CMJ height post exercise, 24 h and 48 h without “post” has been included in this meta-analysis.

**Table 1:** Characteristics of included studies in the systematic review and meta-analysis

Study	*n*	Design	Subjects	Training status	Dose	Intervention	Placebo	Outcome (Δ)
Balshaw et al. [30]	39	RCT	18- to 40-year-old males	Low–moderate PA	15 g	15 weeks RT	Maltodextrin	Young’s modulus (GPa) CP = 0.12 ± 0.22, PLA = 0.17 ± 0.23 PT CSA (mm^2^) CP = 1 ± 3.1, PLA = 1.5 ± 3.2 PT stiffness (N/mm) CP = 242 ± 415, PLA = 322 ± 397
Balshaw et al. [28]	39	RCT	18- to 40-year-old males	Low–moderate PA	15 g	15 weeks RT	Maltodextrin	Muscle volume (cm^3^) Quads + hamstrings + gluteus maximus: CP = 524.5 ± 226.3, PLA = 418.9 ± 125.4 Quads only CP = 271.7 ± 130.6, PLA = 205.1 ± 90.6 MVC (Nm) KE: CP = 46.3 ± 15.6, PLA = 49.7 ± 22.8 KF: CP = 21.9 ± 12.5, PLA = 20.1 ± 13.6 KE 1RM (kg) CP = 14.8 ± 5.6, PLA = 14.2 ± 6
Centner et al. [50]	30	RCT	60.1 ± 7.6-year-old males	1 h/week	15 g	8 weeks BFR	Silicea	Muscle CSA (cm^2^) CP = 9.56 ± 4.27, PLA = 8.91 ± 4.28 Leg strength (N) CP = 108.7 ± 315, PLA = 51.2 ± 139.4
Jendricke et al. [33]	77	RCT	18–50-year-old females	Untrained	15 g	12 weeks RT	Silicea	FFM (kg) CP = 1 ± 0.89, PLA = 0.38 ± 0.87 Leg strength (N) CP = 250.8 ± 147.4, PLA = 233.6 ± 182.9 Handgrip strength (kg) CP = 2.7 ± 2.1, PLA = 1.3 ± 2.4
Jendricke et al. [23]	59	RCT	18–40-year-old females	Running 2 × /week for 60 min	15 g	12 weeks CT	Silicea	FFM (kg) CP = 0.8 ± 0.9, PLA = 0.3 ± 1 Leg strength (kg) CP = 5 ± 5.2, PLA = 6.6 ± 6.3
Jerger et al. [32]	31	RCT	18–40-year-old males	2 h/week	5 g	14 weeks RT	Maltodextrin	PT CSA (mm^2^) CP = 10.94 ± 6.61, PLA = 6.25 ± 5.87 60% and 70% of PT length CP = 10.73 ± 7.76, PLA = 4.19 ± 6.04 PT stiffness (N/mm) CP = 286.9 ± 394.9, PLA = 292.8 ± 565.7 Leg strength (kg) CP = 54.49 ± 40.27, PLA = 68.05 ± 42.73 KE 1RM (kg) CP = 26.94 ± 11.05, PLA = 27.1 ± 20.58
Jerger et al. [31]	40	RCT	18–40-year-old males	1 h/week	5 g	14 weeks RT	Maltodextrin	Muscle thickness (cm) CP = 0.16 ± 0.12, PLA = 0.06 ± 0.06 AT CSA (mm^2^) CP = 7.4 ± 3.51, PLA = 3.13 ± 2.82 AT stiffness (N/mm) CP = 64.23 ± 107.77, PLA = 139.62 ± 154.3 MVC (Nm) CP = 21.75 ± 19.23, PLA = 25.63 ± 20.42
Jerger et al. [89]	32	RCT	18–40-year-old males	Running 2 × /week for 60 min	15 g	12 weeks CT	Silicea	FFM (kg) CP = 0.2 ± 1.2, PLA = 0.5 ± 1.3
Kirmse et al. [51]	57	RCT	24 ± 3-year-old males	Moderate RT	15 g	12 weeks RT	Silicea	Muscle thickness (mm) VL: CP = 2.66 ± 1.62, PLA = 1.99 ± 1.93 RF: CP = 0.64 ± 1.94, PLA = 0.49 ± 1.82 INT: CP = 1.9 ± 2.19, PLA = 1.03 ± 2.64 FFM (kg) CP = 2.05 ± 1.84, PLA = 0.63 ± 1.31 1RM (kg) SQ: CP = 20.6 ± 11.44, PLA = 14.82 ± 9.22 DL: CP = 21.35 ± 13.98, PLA = 19.4 ± 10.99 BP: CP = 12.85 ± 4.46, PLA = 11.8 ± 5.68 MVC (Nm) CP = 25.24 ± 32.94, PLA = 22.56 ± 24.88
Lee et al. [41]	17	RCT	< 21-year-old females	Elite soccer	30 g (+ 0.5 g vitamin C)	10 weeks soccer-specific + body-weight strength and plyometrics	Maltodextrin + fructose + 0.5 g vitamin C	Young’s modulus (GPa) CP = 0.20 ± 0.15, PLA = 0.07 ± 0.13 Muscle thickness (mm) CP = 0.44 ± 2.15, PLA = 0.18 ± 1.31 PT CSA (mm^2^) CP = 0.15 ± 0.24, PLA = 0.01 ± 0.08 PT stiffness (N/mm) CP = 373 ± 222, PLA = 123 ± 255 MVC (Nm) KE: CP = 16.8 ± 28.1, PLA = 2.2 ± 31.3 KF: CP = − 4.4 ± 14.3, PLA = − 3.3 ± 12.4
Lis et al. 2022 [34]	48	RCT	18–25-year-old males	Collegiate rugby players	20 g (+ 50 mg vitamin C)	3 weeks rugby + RT	Maltodextrin	MIS (%) CP = 7.81 ± 2.6, PLA = 7.09 ± 2.8 SJ (N) CP = 64.9 ± 399.8, PLA = 7.6 ± 270.7
Oertzen-Hagemann et al. [90]	25	RCT	24 ± 2.6-year-old males	Moderate RT	15 g	12 weeks RT	Silicea	FFM (kg) CP = 2.56 ± 2.22, PLA = 0.7 ± 1.14 1 RM (kg) SQ: CP = 26.5 ± 13.2, PLA = 17.6 ± 9.9 DL: CP = 24.5 ± 19.1, PLA = 15.9 ± 13.9 BP: CP = 14 ± 6.6, PLA = 9.8 ± 5.5 R: CP = 13.3 ± 8.7, PLA = 6.1 ± 5.8 MVC (Nm) CP = 29.4 ± 22.9, PLA = 14.5 ± 14.3
Zdzieblik et al. [22]	97	RCT	30–60-year-old males	1 h/week	15 g	12 weeks RT	Silicea	FFM (kg) CP = 3.4 ± 2.5, PLA = 1.8 ± 2.1 Leg strength (N) CP = 163 ± 189, PLA = 100 ± 154 Handgrip strength (kg) CP = 1.2 ± 3.7, PLA = 1.8 ± 5.7
Zdzieblik et al. [24]	53	RCT	> 65-year-old males (with sarcopenia)	< 1 h/week	15 g	12 weeks RT	Silicea	FFM (kg) CP = 4.2 ± 5.1, PLA = 2.9 ± 5.2 MVC (Nm) CP = 16.12 ± 5.1, PLA = 7.38 ± 5.2
Recovery-related studies								
Bischof et al. [35]	55	RCT	18–40-year-old males	3 h/week	15 g	12 weeks CT	Silicea	FFM (kg) CP = 0.98 ± 1.89, PLA = 0.67 ± 2.07 Muscle soreness (mm) Post: CP = − 1.13 ± 2.0, PLA = − 0.99 ± 1.65 24 h: CP = − 2.28 ± 1.97, PLA = − 2.52 ± 2.17 48 h: CP = − 2.45 ± 1.78, PLA = − 2.98 ± 2.51 CMJ (cm) Post: CP = 4.89 ± 3.96, PLA = 3.56 ± 2.69 24 h: CP = 6.47 ± 6.15, PLA = 4.01 ± 3.99 48 h: CP = 6.3 ± 5.32, PLA = 3.48 ± 3.83 MVC (Nm) Post: CP = 46.8 ± 56.4, PLA = 27.5 ± 50.1 24 h: CP = 67.3 ± 67.01, PLA = 43.9 ± 63.2 48 h: CP = 68.1 ± 62.8, PLA = 45.9 ± 57.2
Kuwaba et al. [36]	20	RCT	40–65-year-old males	No current regular exercise	10 g	4 weeks no TR	Dextrin	Muscle soreness (mm) Post: CP = 27.36 ± 24.42, PLA = 39.3 ± 26.8 24 h: CP = 26.75 ± 23.77, PLA = 26.7 ± 19.6 48 h: CP = 22.08 ± 20.7, PLA = 20.8 ± 20.7
Prowting et al. [71]	15	RCT	18–35-year-old males	Resistance trained (3 × /week)	15 g	12 days RT	Cornstarch	(all SDs calculated with corr = 0.5) Muscle soreness (mm) 24 h: CP = 35 ± 18, PLA = 29 ± 26 48 h: CP = 30.6 ± 22.4, PLA = 13.1 ± 16.2 CMJ (cm) 24 h: CP = − 1.26 ± 8.8, PLA = − 4.99 ± 7.7 48 h: CP = − 1.84 ± 8.8, PLA = − 0.21 ± 8.6 MVC (Nm) LQ 24 h: CP = − 6.1 ± 27, PLA = − 8.3 ± 30 LQ 48 h: CP = − 4.1 ± 27, PLA = − 1.8 ± 31 RQ 24 h: CP = − 2.3 ± 21, PLA = − 12.5 ± 29 RQ 48 h: CP = − 2.6 ± 21, PLA = − 1 ± 30
Clifford et al. [37]	24	RCT	~ 24-year-old males	2 days/week	20 g (+ 80 mg vitamin C)	9 days no TR	Maltodextrin	(all SDs calculated with corr 0.5) Muscle soreness (mm) Post: CP = 67.8 ± 40.2, PLA = 82.5 ± 52.2 24 h: CP = 94.5 ± 39.9, PLA = 123.6 ± 31.2 48 h: CP = 78.3 ± 41.2, PLA = 110.3 ± 32 CMJ (cm) Post: CP = − 3.7 ± 8.2, PLA = − 3.1 ± 7.2 24 h: CP = − 4.1 ± 8.6, PLA = − 6.3 ± 7.1 48 h: CP = − 3 ± 8.9, PLA = − 6.6 ± 7.6 MVC (Nm) Post: CP = − 115 ± 91, PLA = − 105 ± 140 24 h: CP = − 93 ± 114, PLA = − 115 ± 157 48 h: CP = − 72 ± 112, PLA = − 98 ± 155
Lopez et al. [38]	8	RCT	18–55 years old, both sexes	Recreationally active	3 g	6 weeks no TR	Cellulose	(all SDs calculated with corr 0.5) Muscle soreness (mm) Post: CP = 1.6 ± 3.9, PLA = 0.6 ± 3.1 48 h: CP = 7.7 ± 12.7, PLA = 3.1 ± 6.8

*RCT* randomized controlled trial, *PA* physical activity, *w* week, *d* day, *RT* resistance training, *CT* concurrent training, *TR* training, *CP* collagen peptide group, *PLA* placebo group, *PT* patellar tendon, *AT* Achilles tendon, *CSA* cross-sectional area, *MVC* maximal voluntary contraction, *KE* knee extension, *KF* knee flexion, *1RM* one-repetition maximum, *FFM* fat-free mass, *VL* vastus lateralis, *RF* rectus femoris, *INT* vastus intermedius, *SQ* squat, *DL* deadlift, *BP* bench press, *R* bent-over row, *MIS* Maximal isometric squat, *SJ* Squat jump, *LQ* left quadriceps, *RQ* right quadriceps, *post/24 h/48 h* change scores referring to values obtained preceding muscle damage induced exercise

### Risk-of-Bias Assessment

To evaluate RCT’s risk of bias, the Physiotherapy Evidence Database (PEDro) scale was used as an equivalent alternative to the Cochrane risk of bias tool [43]. Scores and their respective rank are stated as follows: < 4 are considered “poor,” 4–5 are considered “fair,” 6–8 are considered “good,” and 9–10 are considered “excellent” [44]. A.M.M. and K.B. carried out the methodological assessment of the final included studies. A third reviewer (S.S.) resolved any disagreements if necessary. Studies below a “fair” score (< 6) were excluded from the analysis. Additionally, funnel plots visualized and Egger’s regression test calculated risk of publication bias in “maximal strength.” All other parameters were not suitable for publication bias assessment by means of funnel plots since a minimum of ten studies are required. However, funnel plots for all parameters are provided for additional information (Fig. S1 and Fig. S2 in supplementary file).

### Meta-analysis

Meta-analysis was conducted in Jamovi 2.4.11 (open statistical software) with the “MAJOR” package 1.2.4 (W. Kyle Hamilton) by using the standardized mean difference (SMD) of change scores (post–pre values, resulting in Δmean and ΔSD). Therefore, studies only providing pre/post data without delta (only one study [37]) had to be recalculated, as mixing up change scores and pre/post data is intended only for mean differences (MD) [42]. The recalculation was performed using a correlation coefficient (“corr”) of 0.5 [45] and 0.7 [46] to get the ΔSD with the following formula: ΔSD = √ (SD_pre_^2^ + SD_post_^2^ − 2 × corr × SD_pre_ × SD_post_) [47]. In case the results of using 0.5 and 0.7 were similar, 0.5 was chosen for meta-analysis. A random-effects model was applied within all variables, and effect sizes are constituted as SMD with Δmean ± ΔSD and a 95% confidence interval since some studies varied slightly in outcome scales (e.g., cm^2^ and cm^3^ for muscle architectural purposes). The model estimators DerSimonian–Laird, restricted maximum likelihood, and Paul–Mandel were applied. Since there were no significant differences in outcomes when utilizing them, the restricted maximum-likelihood estimator was employed throughout all analyses. *I*^2^ (25%, 50%, and 75% are considered low, moderate, and high variance, respectively [47]), the Cochran’s *Q* statistic (*p* < 0.05 indicating studies do not share a common effect size [48]), and *τ*^2^ as an indicator of between-study variance were calculated as measures of heterogeneity. The Grading of Recommendations Assessment, Development and Evaluation (GRADE) approach was used to evaluate certainty of evidence [49]. Since all included studies depicted RCTs, grading started at “high” certainty. Outcomes could be downgraded to “very low” certainty due to risk of bias, inconsistency, indirectness, imprecision, and publication bias. Upgrading evidence was not possible in RCTs.

## Results

After duplicate removal, 1097 studies were manually screened, of which 25 appeared to meet eligibility criteria. Due to some limitations such as no training intervention and an insufficient control group design, 19 studies, all of them being RCTs published in 2015–2023, were included in the systematic review (Fig. 1). Six records identified from websites and citation searches were excluded due to aforementioned reasons.

In total, 768 subjects, of which 613 were male and 155 were female, participated in the included studies. Of these 768, 122 completed five studies that collected only recovery-related data. The age of participants ranged from ~ 17 to 65, with one study exclusively including elderly above 65 years [24]. CP and PLA were administered daily (except in [41] where three times a week was planned), amounting between 3 and 30 g with 15 g (12 studies) and with a single serving each day being the most common supplementation pattern. Moreover, vitamin C (50, 80, and 500 mg) was also added in three experiments, since vitamin C may increase collagen synthesis [27]. Two studies recruited highly trained athletes, whereas others included subjects ranging from untrained to exercising 3 h per week or three times per week prior to study commencements. Non-recovery-associated studies typically lasted at least 3 weeks, while the majority of studies performed interventions lasting 12–15 weeks. Out of five recovery-related investigations, three did not prescribe any training at all; they rather supplemented 9–28 days and indirectly measured (via MVC, CMJ, and MS) muscle damage immediately before and several times following muscle damage inducing exercises. The other two included a 3-week RT and a 12-week CT. In the last one, participants had to perform tests both before and after the training intervention [35], which was therefore a “unique” approach in this context. Regarding the type of training intervention, one study held a blood-flow-restriction RT, three a concurrent training (body-weight exercises combined with running), and the rest either a body-weight-specific or barbell- and machine-based RT. Maltodextrin, fructose, dextrin, cornstarch, and cellulose were administered as isocaloric placebos, whereas silicea (nine studies) constituted a non-calorie-matched placebo. Table 2 illustrates the risk-of-bias assessment via PEDro scores. None of the included studies reached a total score below “good” (6–8), which implicates low risk of bias overall. Most of the studies failed to satisfy criteria 8 and 9 (11 and 13 studies, respectively), due to missing intention-to-treat analyses and a dropout rate higher than 15%.

**Table 2:** Risk-of-bias assessment expressed as PEDro scores

Study	1	2	3	4	5	6	7	8	9	10	11	Total
Balshaw et al. [30]	1	1	1	1	1	1	1	0	0	1	1	**8**
Balshaw et al. [28]	1	1	1	1	1	1	1	0	0	1	1	**8**
Bischof et al. [35]	1	1	1	1	1	1	1	0	0	1	1	**8**
Centner et al. [50]	1	1	1	1	1	1	1	0	0	1	1	**8**
Clifford et al. [37]	1	1	1	1	1	1	1	1	1	1	1	**10**
Jendricke et al. [33]	1	1	1	1	1	1	1	1	1	1	1	**10**
Jendricke et al. [23]	1	1	1	0	1	1	1	0	0	1	1	**7**
Jerger et al. [32]	1	1	1	1	1	1	1	0	0	1	1	**8**
Jerger et al. [31]	1	1	1	1	1	1	1	0	0	1	1	**8**
Jerger et al. [89]	1	1	1	1	1	1	1	0	0	1	1	**8**
Kirmse et al. [51]	1	1	1	1	1	1	1	1	0	1	1	**9**
Kuwaba et al. [36]	1	1	1	1	1	1	1	0	0	1	1	**8**
Lee et al. [41]	1	1	0	1	1	1	0	0	0	1	1	**6**
Lis et al. [34]	1	1	1	1	1	1	1	1	0	1	1	**9**
Lopez et al. [38]	1	1	1	1	1	1	1	1	1	0	1	**9**
Oertzen-Hagemann et al. [90]	1	1	1	1	1	1	1	1	1	1	1	**10**
Prowting et al. [71]	1	1	1	1	1	1	1	1	1	1	1	**10**
Zdzieblik et al. [22]	1	1	1	1	1	1	1	0	1	1	1	**9**
Zdzieblik et al. [24]	1	1	1	1	1	1	1	1	0	1	1	**9**

Total scores are in bold. Also include definitions: 1 = criterion satisfied, 0 = not satisfied. Rating: < 4 “poor”, 4–5 “fair”, 6–8 “good”, 9–10 “excellent” (the higher, the better). Criterions 1-11 are listed. Cristerion 1 is mandatory, but is usually not included in the calculation

**Fig. 2:**
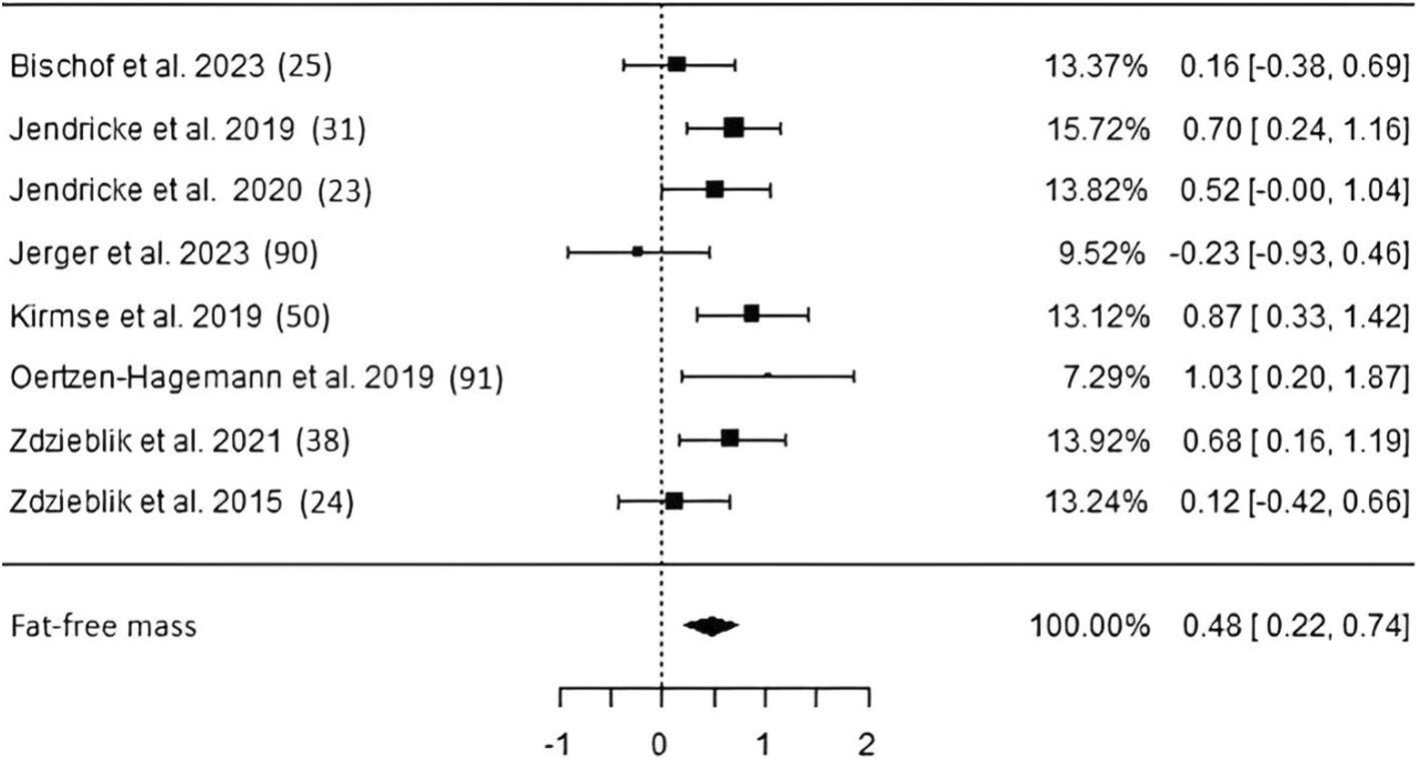
Studies investigating fat-free mass with individual weights, SMDs (as filled squares), overall SMD (as filled rhombus), and confidence intervals

*Fat-free mass* Eight studies including 418 subjects investigating fat-free mass changes following long-term CP supplementation (Fig. 2) revealed a significant pooled effect size (ES) of 0.48 [*p* < 0.01, confidence interval (CI) 0.22–0.74]. Two studies used DXA [22, 24], and six remaining studies chose BIA to obtain body composition. Low heterogeneity along with low risk of publication bias has been detected (Table 3).

**Table 3:** Estimated SMD, heterogeneity statistics and publication bias assessment (by means of Egger’s regression test)

Parameter	Estimate	*p*	*τ*^2^	*I*^2^ (%)	*Q*	*p* (*Q*)	Egger’s regression test (*p*)
Fat-free mass	0.48	** < 0.001**	0.054	40	12.237	0.093	0.924
Tendon morphology	0.672	**0.011**	0.19	55.52	9.217	0.056	0.141
Tendon mechanical properties	0.049	0.822	0.126	45.37	9.446	0.093	0.011
Muscle architecture	0.387	** < 0.001**	0	0	4.985	0.662	0.593
Maximal strength	0.192	**0.002**	0	0	25.701	0.48	0.615
Recovery maximal strength post	0.145	0.474	0	0	0.791	0.673	0.486
Recovery maximal strength 24 h	0.26	0.144	0	0	0.622	0.961	1
Recovery maximal strength 48 h	0.306	0.087	0	0	2,114	0.715	0.211
Recovery reactive strength 24 h	0.414	0.051	0	0	0.166	0.920	0.806
With corr 0.7	0.452	**0.033**	0	0	0.105	0.949	0.988
Recovery reactive strength 48 h	0.427	**0.045**	0	0	1.75	0.417	0.232
Recovery muscle soreness post	− 0.208	0.256	0	0	1.217	0.749	0.791
Recovery muscle soreness 24 h	− 0.049	0.785	0	0	3.447	0.328	0.489
Recovery muscle soreness 48 h	0.093	0.703	0.117	40.79	6.78	0.148	0.715

Statistically significant results are in bold

**Fig. 3:**
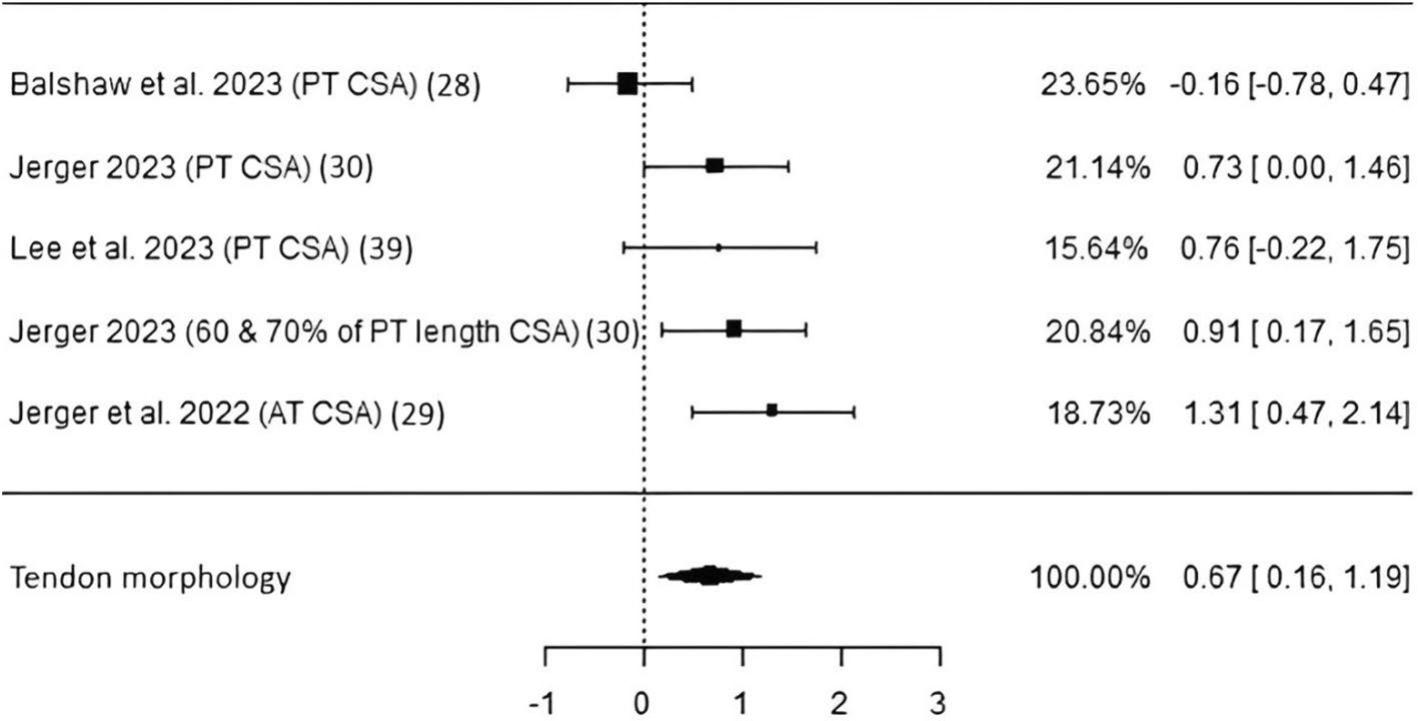
Studies investigating tendon morphology with individual weights, SMDs (as filled squares), overall SMD (as filled rhombus), and confidence intervals. *PT* patellar tendon, *AT* Achilles tendon, *CSA* cross-sectional area

*Tendon morphology* Four studies including 114 subjects obtained data on patellar and Achilles tendon cross-sectional area by using either magnetic resonance imaging (MRI) [30, 32] or ultrasound (US) [31, 41]. A significant pooled ES of 0.67 (*p* = 0.01, CI 0.16–1.19) with moderate heterogeneity and low risk of bias was found (Table 3, Fig. 3). It is noteworthy that when Jerger et al. [32] was excluded, where 60% and 70% of the PT CSA was also measured and data are provided in the article, the random-effects model would not reach statistical significance (*p* = 0.058).

**Fig. 4:**
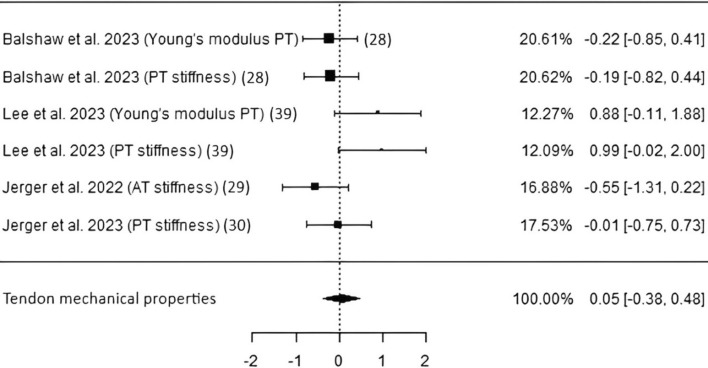
Studies investigating tendon mechanical properties with individual weights, SMDs (as filled squares), overall SMD (as filled rhombus), and confidence intervals. *PT* patellar tendon, *AT* Achilles tendon

*Tendon mechanical properties* Four studies, including 111 participants from which data of Young’s modulus and stiffness of either PT or AT by means of sonography were acquired, resulted in a nonsignificant pooled ES of 0.05 (*p* = 0.82, CI − 0.38 to 0.48) as shown in Fig. 4. Heterogeneity appeared to be low, and Egger’s regression test hinted potential publication bias (*p* = 0.01; Table 3). Moreover, Ref. [41], having a PEDro score of merely 6 and therefore being the only study with the lowest score within the systematic review and meta-analysis, apparently causes overall heterogeneity.

**Fig. 5:**
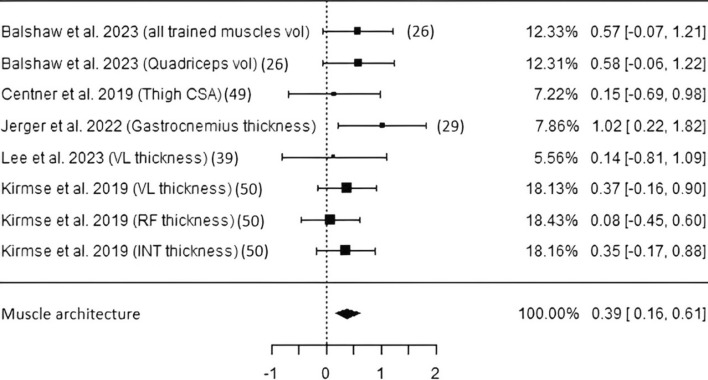
Studies investigating muscle architecture with individual weights, SMDs (as filled squares), overall SMD (as filled rhombus), and confidence intervals. *Vol* volume, *CSA* cross-sectional area, *VL* vastus lateralis, *RF* rectus femoris, *INT* vastus intermedius

*Muscle architecture* Five studies including 161 subjects investigated muscular adaptations using either MRI [28, 50] or sonography [31, 41, 51]. As seen in Fig. 5, CP intake led to a statistically significant pooled estimate (ES 0.39, *p* < 0.01, CI 0.16–0.61). Risk of bias as well as heterogeneity were of low potential/values (Table 3 and Fig. S1).

**Fig. 6:**
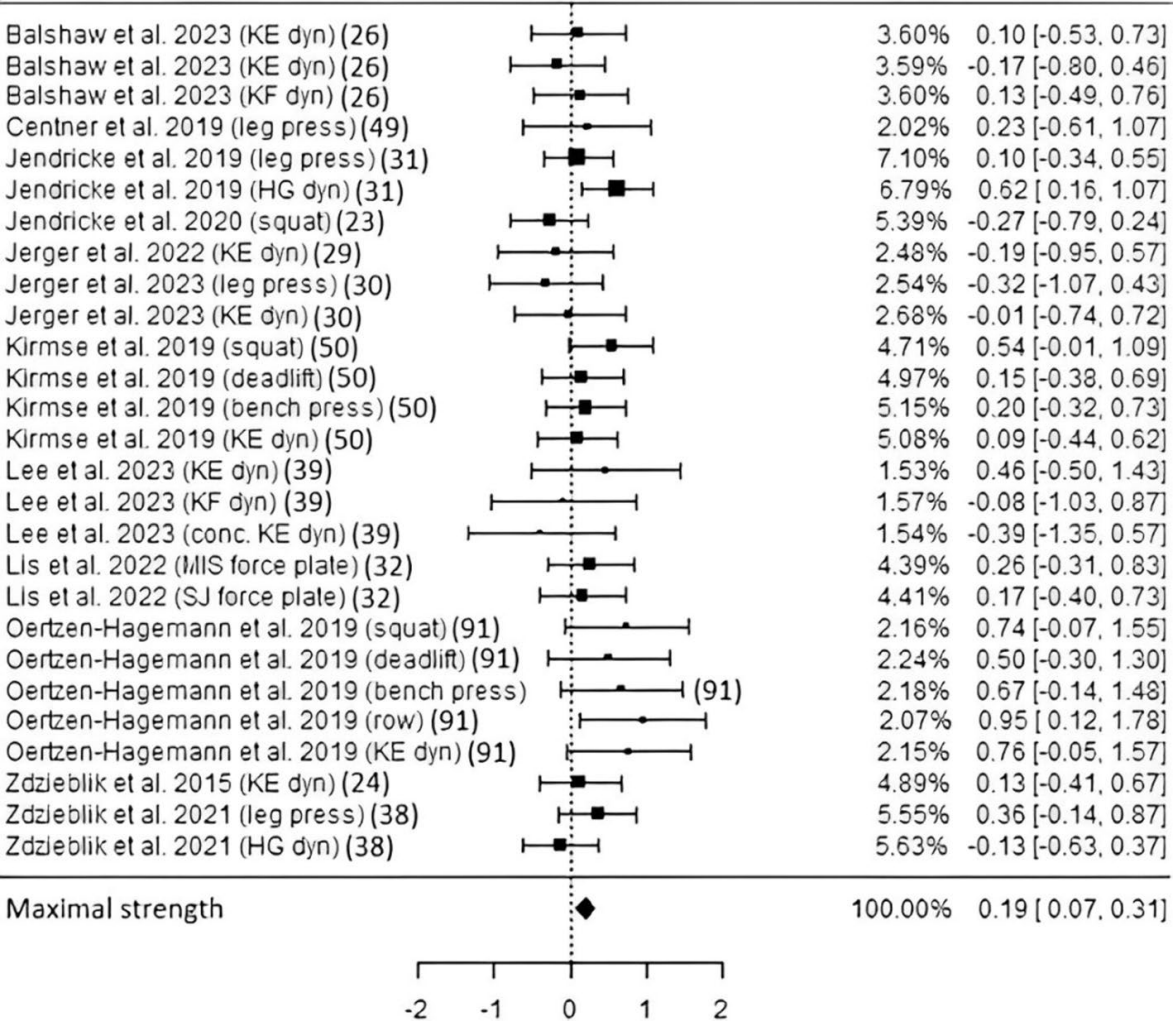
Studies investigating maximal strength with individual weights, SMDs (as filled squares), overall SMD (as filled rhombus), and confidence intervals. The first two values differed in units and instructions, but both derived from using a dynamometer. *KE* knee extension, *KF* knee flexion, *dyn* dynamometer used, *HG* handgrip, *conc.* concentric, *MIS* maximal isometric squat, *SJ* squat jump

*Maximal strength* Eleven studies including about 511 subjects (depending on selected parameters) resulted in a significantly higher maximal strength following CP supplementation (ES 0.19, *p* < 0.01, CI 0.07–0.31; Fig. 6) compared with placebo. Maximal strength was measured by means of a dynamometer, leg press, multijoint resistance exercise (weights), or a force plate. Heterogeneity and risk of bias appeared to be low (Table 3, Fig. S1). The exclusion of the 3-week trial by Lis et al. 2022 [34] led to similar gains in maximal strength (ES 0.19, *p* < 0.01, CI 0.06–0.32), indicating long-term adaptation elicited by CP administration.

**Fig. 7:**
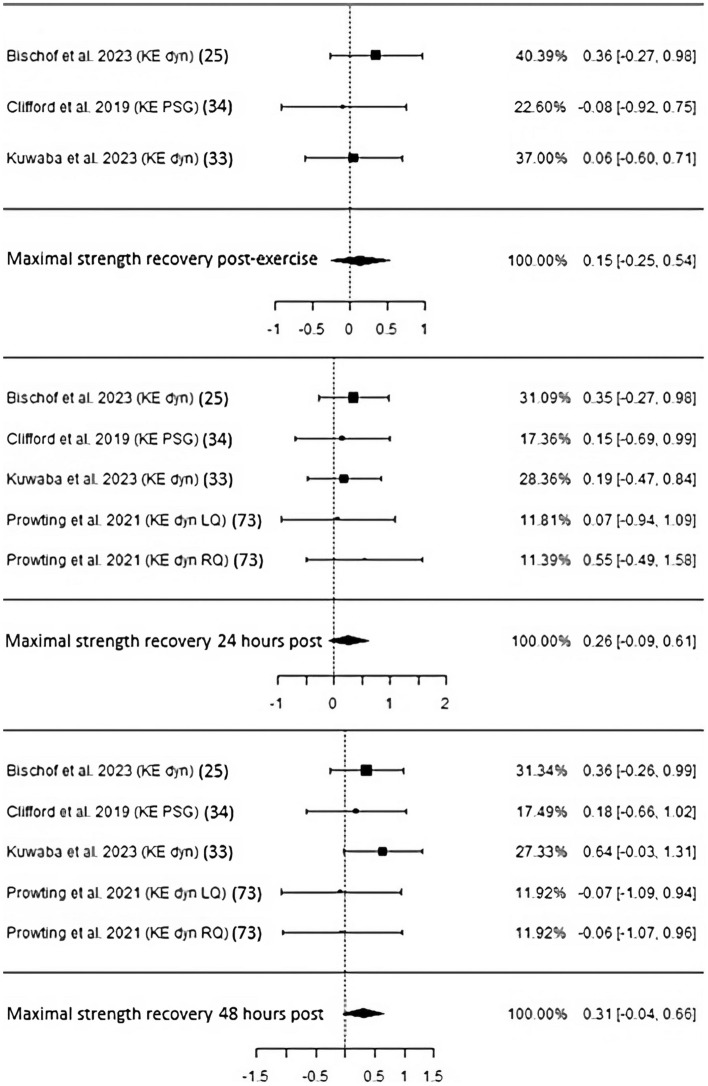
Studies investigating maximal strength recovery immediately post, 24 h, and 48 h following exercise-induced muscle damage with individual weights, SMDs (as filled squares), overall SMD (as filled rhombus), and confidence intervals. Studies investigating with individual weights, SMD and confidence intervals. PSG, portable strain gauge

*Recovery of maximal and reactive strength and muscle soreness* For immediate regeneration of maximal strength after exercise-induced muscle damage, three studies with 98 participants were not able to reach statistical significance (ES 0.15, *p* = 0.47, CI − 0.25 to 0.54; Fig. 7). Both heterogeneity and publication bias were of low values (Table 3). Twenty-four hours following muscle damaging exercise(s) that either comprised several sets of muscle lengthening drop jumps [35, 37], squats [36] or an upper-body resistance training [38] did not lead to statistical significance (ES 0.26, *p* = 0.14, CI − 0.09 to 0.61). Four studies including 113 participants overall presented low risk of bias and heterogeneity. The same number of experiments and subjects also failed to reach significance (ES 0.31, *p* = 0.09, CI − 0.04 to 0.66). Heterogeneity and the potential of risk of bias stayed low.

**Fig. 8:**
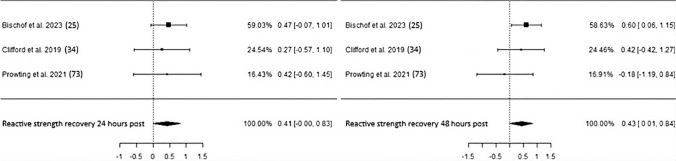
Studies investigating reactive strength recovery 24 and 48 h following exercise-induced muscle damage with individual weights, SMDs (as filled squares), overall SMD (as filled rhombus), and confidence intervals

Regarding recovery of reactive strength measured by countermovement jumps 24 h after intense exercise bouts, three studies totaling 91 participants either led to or did not show statistical significance depending on the chosen correlation coefficient (“corr”) used to calculate ΔSD when data provision was insufficient (Fig. 8, Table 3). Accordingly, a corr of 0.7 resulted in an ES of 0.45 and *p*-value of 0.03, whereas a corr of 0.5 resulted in a nonsignificant *p*-value of 0.05 with an ES of 0.41. Low heterogeneity and no risk of bias were identified in both cases. CP administration also improved reactive strength recovery 48 h following exercise (ES 0.43, *p* = 0.045, CI 0.01–0.84) with low heterogeneity and risk of bias.

**Fig. 9:**
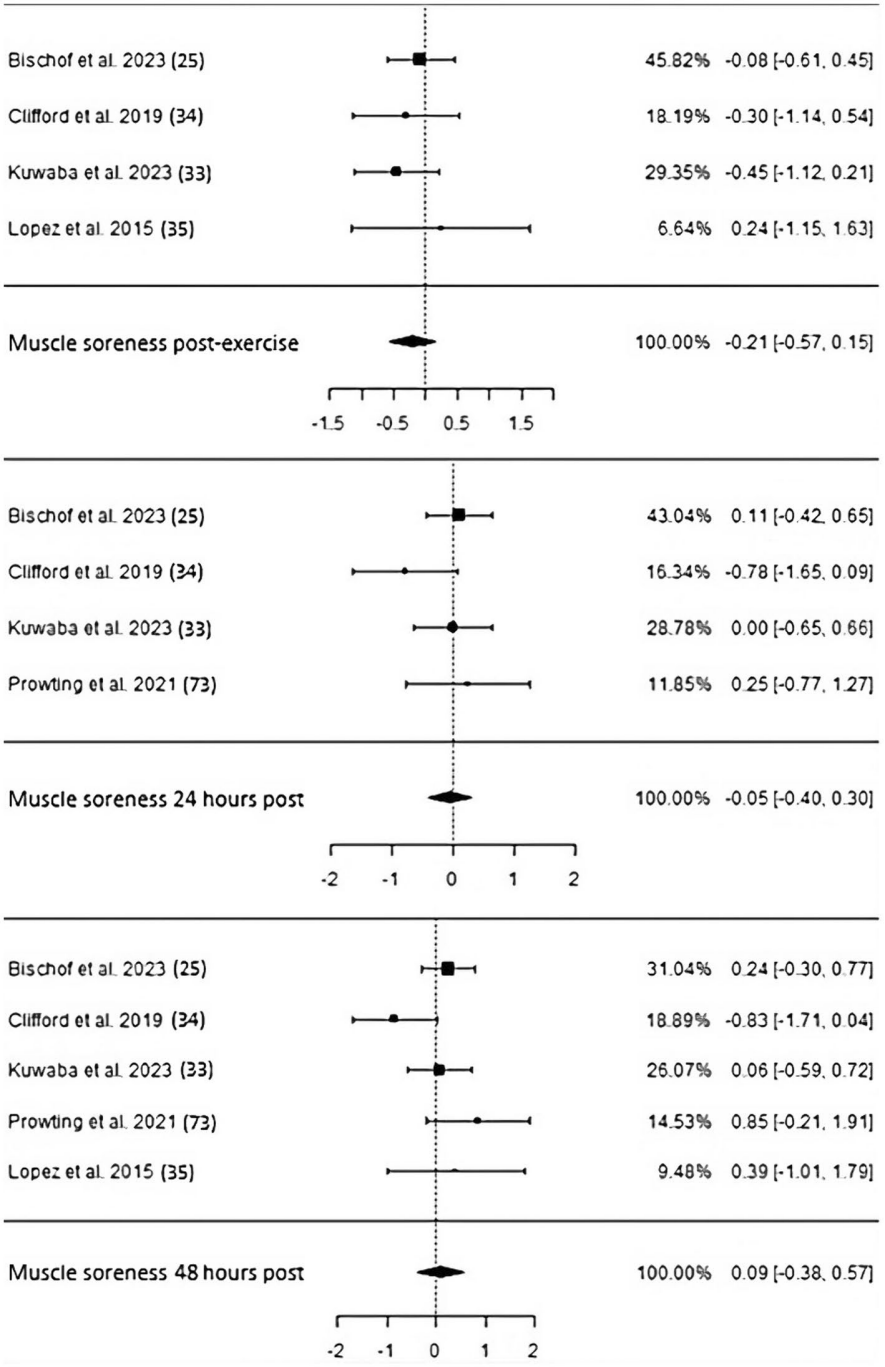
Studies investigating muscle soreness post, 24 h, and 48 h following exercise-induced muscle damage with individual weights, SMDs (as filled squares), overall SMD (as filled rhombus), and confidence intervals

Muscle soreness typically evaluated by means of a visual analog scale has not been significantly influenced by CP supplementation in four studies comprising 121 subjects, neither immediately post nor 24 h (4 studies, 127 subjects) and 48 h (5 studies, 135 subjects) after muscle-damaging exercise (post: ES − 0.21, *p* = 0.26, CI − 0.57 to 0.15; 24 h: ES − 0.05, *p* = 0.79, CI − 0.4 to 0.3; 48 h: ES 0.09, *p* = 0.7, CI − 0.38 to 0.57; Fig. 9). Heterogeneity continuously stayed low, and no serious potential of risk of bias emerged (Table 3).

*Certainty of evidence* Table 4 illustrates the certainty of evidence for each outcome with additional information regarding reasons for downgrading. Fat-free mass can be considered moderate; certainty of evidence of muscle architecture, maximal strength as well as maximal & reactive strength and muscle soreness following exercise-induced muscle damage recovery appears to be low. Very low certainty of evidence exists for tendon mechanical properties and tendon morphology.

**Table 4:** Certainty of evidence for each outcome

Outcome	No. of studies	No. of participants	Estimated effect (as SMD)	Certainty of evidence (GRADE)
Fat-free mass	8	418	0.48	⨁⨁⨁◯ moderate^d^
Tendon morphology	4	114	0.67	⨁◯◯◯ very low^b,c,d^
Tendon mechanical properties	4	111	0.05	⨁◯◯◯ very low^a,c,d,e^
Muscle architecture	5	161	0.39	⨁⨁◯◯ low^c,d^
Maximal strength	11	511	0.19	⨁⨁◯◯ low^c,d^
Recovery maximal strength post	3	98	0.15	⨁⨁◯◯ low^c,d^
Recovery maximal strength 24 h	4	113	0.26	⨁⨁◯◯ low^c,d^
Recovery maximal strength 48 h	4	113	0.31	⨁⨁◯◯ low^c,d^
Recovery reactive strength 24 h	3	91	0.41	⨁⨁◯◯ low^c,d^
Recovery reactive strength 48 h	3	91	0.43	⨁⨁◯◯ low^c,d^
Recovery muscle soreness post	4	121	− 0.21	⨁⨁◯◯ low^c,d^
Recovery muscle soreness 24 h	4	127	− 0.05	⨁⨁◯◯ low^c,d^
Recovery muscle soreness 48 h	5	135	0.09	⨁⨁◯◯ low^c,d^

Reasons for downgrades: a = risk of bias, b = inconsistency/heterogeneity, c = participants and/or interventions differed (training history, duration, study design), d = imprecision/ wide confidence intervals, e = publication bias (detected via Egger’s test)

*SMD* standardized mean difference

## Discussion

To the best of our knowledge, this is the first systematic review and meta-analysis investigating the effects of longer-term collagen peptide (CP) supplementation in combination with regular physical training on strength, musculotendinous remodeling, functional recovery, and body composition in healthy adults.

Our findings indicate that prolonged CP intake may substantially enhance fat-free mass (FFM) and improve tendon morphology, muscle architecture, maximal strength, and recovery in reactive strength following multiple bouts of muscle-lengthening exercises. However, it is worth noting that the certainty of evidence for any parameter was not evaluated as high.

In line with the findings of two systematic reviews [39, 52], significant favorable body composition changes have been observed following several weeks of consuming CP together with either resistance (RT) or concurrent (CT) training with a moderate certainty of evidence. Furthermore, the current meta-analysis demonstrated enhanced fat-free mass (FFM) along with muscle architectural adaptations, expressed as augmentations in one-, two-, and three-dimensional measurements. Both body-weight and conventional barbell-assisted resistance training (RT) have been shown to stimulate muscle protein synthesis, an effect that is further amplified when additional protein is ingested promptly [53]. Typically, an amino acid composition high in essential amino acids is thought to be crucial for maximizing muscle gains. Leucine, a branched-chain amino acid, is particularly notable for its myocellular signaling ability, enhancing the phosphorylation status of p70S6k, mTOR, and 4E-BP1 [54]. This has led to increases in satellite cell numbers and protein expression of MyoD and myogenin—proteins involved in myoblast differentiation, myogenesis, and the regulation of anabolic balance—when combined with RT in mice [55]. Despite being low in leucine and high in glycine, proline, and hydroxyproline, CPs have also demonstrated the ability to contribute to an increase in FFM. This effect is supported by a moderate level of evidence (one level deducted due to wide ranges of effects) in the current meta-analysis, partly contrasting with previous results.

The present analysis has unveiled a significant increase in muscle mass accrual, measured by thickness, diameter, and volume, albeit with a low certainty of evidence. This low certainty was primarily attributed to indirectness, stemming from differing populations in terms of training status, and imprecision, evidenced by wide confidence intervals. Analogous to leucine, a single portion of CP has been shown to evoke a higher upregulation of key anabolic pathways (PI3K-Akt, MAPK) involved in myofibrillar protein synthesis 4 h following RT [25]. Moreover, an in vitro experiment treating C2C12 cells with the dipeptide hydroxyprolyl-glycine elicited phosphorylation of p70S6k, mTOR, and Akt, indicating myogenic differentiation [56]. So far, only one RCT compared the hypertrophic effects of whey protein and leucine-matched CPs. After 10 weeks of resistance training and daily doses of 35 g/day, whey protein, compared with CP, significantly increased muscle mass in the vastus lateralis and biceps brachii of previously untrained individuals. This suggests that protein sources rich in essential amino acids, like whey, are more effective than CPs for increasing muscle thickness [57]. Additionally, it is worth mentioning that the studies primarily focused on muscle groups in the lower limbs, with a noticeable lack of data on muscle adaptation in the upper limbs. Future experiments should consider the involvement of upper-body muscles, as varying inter-muscle mass adaptability has been observed in several long-term exercise trials [58–61]. Therefore, it remains to be determined whether CP intake also influences muscle growth in the upper body.

In line with the current findings demonstrating a small yet significant overall benefit from CP supplementation on maximal strength, it is commonly observed that muscle mass accrual is associated with enhanced strength [62]. This association can be attributed to the fact that myofibrils are the primary force-producing units within the musculoskeletal system. Additionally, factors such as muscle fiber composition, muscle architecture, neural activation of agonist and antagonist muscles, and specific tension also contribute to increases in muscle strength. Balshaw and colleagues presented significantly higher evoked contractile twitch peak torque after chronic CP intake but no group-specific quadriceps tension and muscle architectural differences. Only the pennation angle of the vastus intermedius (among all other quadriceps muscles) showed a tendency to have increased over a 15-week RT period [28]. Furthermore, the results of another study failed to demonstrate a relative and absolute muscle fiber type and group specific adaptation following 15 g CP together with 12 weeks of RT, possibly justified by the already RT experienced cohort and their already high protein intake of > 1.7 g/kg/day. The authors hypothesized that the observed changes were more likely due to adaptations in passive tissue components rather than myofibrillar accrual [51]. Nevertheless, another 12-week RCT investigating older sarcopenic men undergoing RT plus daily 15 g of CP led to significantly higher strength output in the CP group, outlining CPs’ potential effects in non-RT-trained individuals [24].

As shown in the current meta-analysis, tendon CSA (mostly comprising patella tendons) was significantly increased following long-term RT with CP intake, indicating a pronounced tendon remodeling particularly after several months of exercising. This is in accordance with a comprehensive systematic review and meta-analysis highlighting most beneficial morphological tendon changes after 12-week training interventions, regardless of muscle contraction types (concentric, isometric, eccentric) [63]. The potential superiority of CP supplementation might lie in the enhanced collagen synthesis of force-transmitting passive tissues. Blood sera from subjects who performed 6-min rope-skipping sessions combined with 15 g of gelatin have been shown to significantly increase collagen concentration in human-engineered ligaments compared with 5 g and a placebo. This increase was accompanied by elevated levels of the N-terminal peptide of pro-collagen I (P1NP), a marker of collagen formation. In parallel, collagen-specific amino acids glycine, proline, hydroxyproline, and hydroxylysine were significantly elevated up to 2 h after gelatin ingestion in the subjects’ blood [7]. Absorption experiments in human blood have also affirmed rising levels of collagen-specific amino acids, di- and tripeptides [8, 64, 65], achieving even higher uptakes when ingesting low-molecular-weight peptides (~ 3 kDa and below) derived from collagen hydrolysates [66]. PEPT-1, an active transmembrane peptide transporter located at the intestinal luminal brush border, may be responsible for the uptake of di- and tripeptides, and some of them, containing hydroxyproline and/or proline, might withstand cytosolic peptidases so that intact entry into the blood stream is feasible [5]. Lastly, since high loads from physically demanding activities apply enormous tendon stress, a large tendon CSA may act in an injury-preventive manner from a physical point of view (stress = force/CSA) [32]. Based on the current findings, CP supplementation might help adolescent athletes balance out muscle and tendon disparities by promoting tendon CSA growth to match muscle growth that is typically seen in adults [67]. However, the level of evidence appears to be very low, elicited by concerns of heterogeneity, study population differences, and wide effect ranges.

Somewhat surprisingly, tendon mechanical properties revealed no difference between CP and placebo in the present analysis although significant morphological adaptations occurred. Tendon stiffness (ratio of tendon elongation to tendon force applied) and Young’s modulus (stiffness multiplied by the ratio of tendon length over CSA) as parameters defining tendon mechanical properties in the present analysis have already been reported to be significantly increased with no concomitant changes in tendon CSA [68]. A recent systematic review and meta-regression also revealed that an increased modulus rather than the CSA was the predominant moderator of enhanced tendon stiffness. The main reason for the rise in tendon stiffness due to training is thought to be an increase in gene expression related to anabolic responses to strain, leading to, seemingly lower, collagen synthesis and turnover. Additionally, enhanced enzymatic cross-linking of collagen might also play a role [69] as well as noncollagenous extracellular matrix (ECM) components (e.g., glycoproteins, elastin) which may reduce energy loss and help recover stored energy [70]. Stiffness and Young’s modulus are supposed to be highly dependent on load intensity and contraction type, with eccentric ones being superior [68]. In one out of four included studies in the current meta-analysis, subjects performed body-weight and plyometric exercises instead of high-load RT [41]. In addition, two of four studies recruited already (resistance) training-experienced individuals [30, 41], emphasizing the challenges in identifying significant effects attributed to a well-developed training level.

Muscle soreness as a frequently used indicator of muscle damage was usually gauged several times within 48 h following muscle damage-inducing exercise (MDIE) in the current meta-analysis without any significant effects. Participants of included studies documented their perceived muscle soreness immediately following either countermovement jumps (CMJ) or squats (of note, two [38, 71] out of five studies did not provide this information). Activity-related pain could have influenced the results of CMJ or squat exercises, both of which involve a wide range of motion. CP has already been effectively utilized to notably decrease pain during physical activity after a 12-week regimen of 5 g of CP daily in athletes experiencing exercise-related knee pain [72, 73]. However, there was no significant difference in muscle soreness reported, indicating that only subjective pain was recorded, which may not be influenced by long-term CP intake. As muscle soreness is also closely related to inflammation [74] and expresses the degree of muscle damage and/or regeneration at least to some extent [75], animal studies have demonstrated anti-inflammatory potential of CPs and glycine alone. CPs were able to positively modulate local and systemic inflammatory response by reducing swelling and interleukin-6 (IL-6)-mediated lipopolysaccharide (LPS) production, possibly via the activation of glycine-gated chloride channels [76]. A solely glycine administration also has been reported to decrease plasma and mRNA tumor necrosis factor-α (TNF-α) levels as well as reduced muscle mRNA expression of Toll-like receptor 4 (TLR4) and nucleotide-binding oligomerization domain protein 2 (NOD2), two major proteins involved in inflammatory signal transduction [77]. In a short-term human RCT, a 1-week daily CP supplementation was not able to alter IL-6 levels compared with a placebo following several bouts of muscle-damaging exercises [37]. Therefore, it remains to be investigated if and how CPs could influence human muscle soreness.

In the present analysis, elevated regeneration of maximal strength for each time point (post, 24 h, 48 h) lacked statistical significance indicating no influence of CP intake in this respect. Performed tests (biomechanical-related exercises such as CMJ or knee extensions performed on a force-capturing dynamometer) typically lasted seconds and subjects required 0.5–2 min of rest. Considering methodological aspects, a study conducted on minimal rest period between maximal voluntary contractions concluded that a 60-s rest interval is sufficient but seems mandatory for the regeneration of force-producing capacity in healthy individuals [78]. A shorter rest period thus might have influenced the outcome. Reactive strength (measured by means of CMJ height) displayed a tendency to reach significant effects at 24 h (depending on the correlation coefficient used to calculate ΔSD) and eventually did at 48 h (ES 0.43, *p* = 0.045), which underlines a certain correlation between these two types (maximal and reactive) of muscle strength [79]. The results might be difficult to interpret since one investigation sought to determine differences before and following an implemented 12-week CT [35] whereas others did not include any training intervention and therefore administered CPs to a lesser time extent (1–6 weeks). One possible explanation of CPs showing a positive trend/effect here appears to be the chronic adaptation of tissues that possess the capability to store and release elastic energy during muscle contraction such as actin and myosin filaments and their formed cross-bridges, titin, and the connective tissue scaffolding of the ECM. Regarding tendons, it is known that the retention and release of elastic energy can significantly influence the force, power, and velocity of movement [80]. In general, the ECM is naturally stiffer than muscle fibers and plays a significant role in generating passive force within muscles. Hence, the observed effects of greater improvements in reactive strength compared with maximal strength in the current meta-analysis could be attributed to the positive influence of the stretch–shortening cycle during countermovement jumps (CMJ), a factor less influential during maximum voluntary contraction tests [71]. Another speculative reason for enhancing performance regenerative capacity could be attributed to the reduction in oxidative stress and inflammation associated with CPs. Glycine (the most abundant amino acid in CPs) acts through glycine receptors located on several immune-related cell types. It has been reported to attenuate superoxide production in neutrophil granulocytes [81] and may decrease the synthesis of pro-inflammatory mediators while inhibiting LPS-induced cytokine production [82]. Recently, CP’s potential ability to reduce endotoxemia [measured via lipopolysaccharides (LPS)] after gastrointestinal stress induced by intense aerobic exercise has been demonstrated in humans [83]. A recent review also revealed hydrolyzed CP to have possible anti-inflammatory and chondroprotective effects in patients with osteoarthritis, but considerable limitations exist due to methodological disparities [84]. Furthermore, most of the supplementation interventions lasted > 3 months, whereas the periods in the current analysis regarding strength regeneration were of much shorter durations. It remains to be elucidated which underlying factors and mechanisms led to such CP associated short-term adaptations since force transmitting tissues (e.g., intramuscular connective tissue and tendons) are unable to adapt within a week. Also, significantly increased collagen formation accompanied by several days of CP intake has not been reported (supported by blood levels of P1NP [37]).

*Strengths and limitations* One of the major strengths in this review demonstrates the overall low risk of bias with no studies having ranked less than “good” (PEDro score). However, future investigations are encouraged to conduct additional intention-to-treat analyses and report end-of-study (“post-intervention”) data of as many participants starting the trial as possible to further diminish risk of bias. In addition, most of the trials (12 out of 19) administered 15 g CP that is currently expected to elicit the most beneficial sports performance and injury-preventive results. Nonetheless, a recently published study suggests to provide 30 g instead of 15 g CP to further enhance collagen synthesis response, at least in resistance-trained subjects following an acute RT bout [85], also highlighting the urge for CP dose–response experiments. Based on previous investigations applying varying protein dosages in active individuals, at least muscle protein synthesis has been significantly increased using more than 15 g (20 g after RT [86] and ~ 0.5 g/kg body weight following endurance training [87]), surmising that also greater doses (> 15 g) of CP are required to act maximally stimulating in experienced athletes.

Although statistical effects throughout this meta-analysis have been identified, the certainty of evidence using the GRADE approach was moderate to very low for significant outcomes. This uncertainty stemmed from several factors, including the indirectness of evidence, as subjects sometimes differed in their training status, resulting in heterogeneity in musculoskeletal adaptability. Imprecision of results through “unfavorable” distribution of confidence intervals, risk of bias, heterogeneity (as indicated by a higher *I*^2^), differing types of intervention, and their length (in recovery-associated studies) and potential publication bias as indicated by a significant Egger’s test can also be constituted as downgrades. Apart from that, not all authors were able to share change scores of the standard deviation of their trials and respective subjects that consequently caused using a specific correlation coefficient (“corr”) to calculate the ΔSD of three studies. Depending on the corr, the results changed markedly, as demonstrated by the significant recovery in reactive strength observed 24 h post-exercise (corr 0.5 → *p* = 0.051; corr 0.7 → *p* = 0.033). Overall, the number of studies included for tendon- and recovery-related parameters was low, indicating further future research in this field.

The magnitude of musculoskeletal adaptability is highly dependent on individual training history as well as age. Two included studies were completed by older subjects (60 +), with one even recruiting sarcopenic elderly subjects, which might have influenced at least some outcomes. Moreover, the integration of highly trained athletes involved in two trials might have left some impact on the results since musculotendinous remodeling happens at lower rates compared with untrained/recreationally active subjects, therefore possibly reducing effects of the intervention. Lastly, three experiments added additional vitamin C to CP supplementation, expecting to increase hypothetical adaptational processes in collagen-containing tissues, given that vitamin C is an essential cofactor of lysyl hydroxylase and prolyl hydroxylase, both indispensable for collagen biosynthesis [88]. However, there has not been any CP + training intervention trial conducted so far that compared both CP + vitamin C and CP alone.

## Conclusion

Prolonged collagen peptide (CP) supplementation combined with resistance or concurrent training appears to be a beneficial adjunct for healthy active adults and athletes striving for an increase in fat-free mass and maximal strength and an improvement in tendon morphological properties and reactive strength recovery. CP’s potential ability to further increase tendinous cross-sectional area may represent an effective option to anticipate sports-related tendon injuries. The current rationale to induce aforementioned adaptations seems to be a daily dose of 15 g CP for at least 8 weeks. Acute supplementation patterns (1–6 weeks) could also aid in enhancing regenerative capacity, but underlying mechanisms behind possible effects remain unclear. Future investigations are encouraged to figure out the optimal CP dosage and composition as well as their effective absorption and incorporation into force-producing and transmitting tissues. Moreover, experienced athletes might profit from higher CP (> 15 g) dosages as recently reported in other trials investigating protein supplementation and its effect on enhanced protein synthesis. In particular, individuals participating in sports known to exert significant stress on tendons (such as powerlifting and team sports involving uncontrolled movements like soccer and tennis) could potentially derive the greatest benefit from several months of collagen peptide use.

## Supplementary Information

Below is the link to the electronic supplementary material.

Supplementary file1 (PDF 596 kb)

## Declarations

### Availability of data and material

The raw data supporting the conclusions of this article will be made available by the authors, without undue reservation upon request.

### Code availability

No self-written code was used for the meta-analysis. The freeware Jamovi for Microsoft Windows 11 (https://www.jamovi.org/download.html) performed all meta-analyses and created all forest plots.
