# Supplementary material for: Impact of Collagen Peptide Supplementation in Combination with Long-Term Physical Training on Strength, Musculotendinous Remodeling, Functional Recovery, and Body Composition in Healthy Adults: A Systematic Review with Meta-analysis
Source: Sports Med. 2024 Jul 26;54(11):2865–88. doi: 10.1007/s40279-024-02079-0 (PMC11561013; doi:10.1007/s40279-024-02079-0)
Supplement: Supplementary file 1 — Supplementary file1 (PDF 596 kb) [file 40279_2024_2079_MOESM1_ESM.pdf]

Impact of collagen peptide supplementation in combination with long-term physical training on strength, musculotendinous remodeling, functional recovery and body composition in healthy adults – A systematic review with meta-analysis

Kevin Bischof [2,4,*], Anna Maria Moitzi [1,4], Savvas Stafilidis [3], Daniel König [1,2,4]

[1] Faculty of Life Sciences, Department for Nutrition, Section for Nutrition, Exercise and Health, University of Vienna, Vienna, Austria

[2] Centre for Sports Science and University Sports, Department of Sports Science, Section for Nutrition, Exercise and Health, University of Vienna, Vienna, Austria

[3] Centre for Sports Science and University Sports, Department for Biomechanics, Kinesiology and Computer Science in Sport, University of Vienna, Vienna, Austria

[4] Vienna Doctoral School of Pharmaceutical, Nutritional and Sport Sciences, University of Vienna, Vienna, Austria

Journal Name: Sports Medicine

*** Correspondence:**
Kevin Bischof
kevin.bischof@univie.ac.at

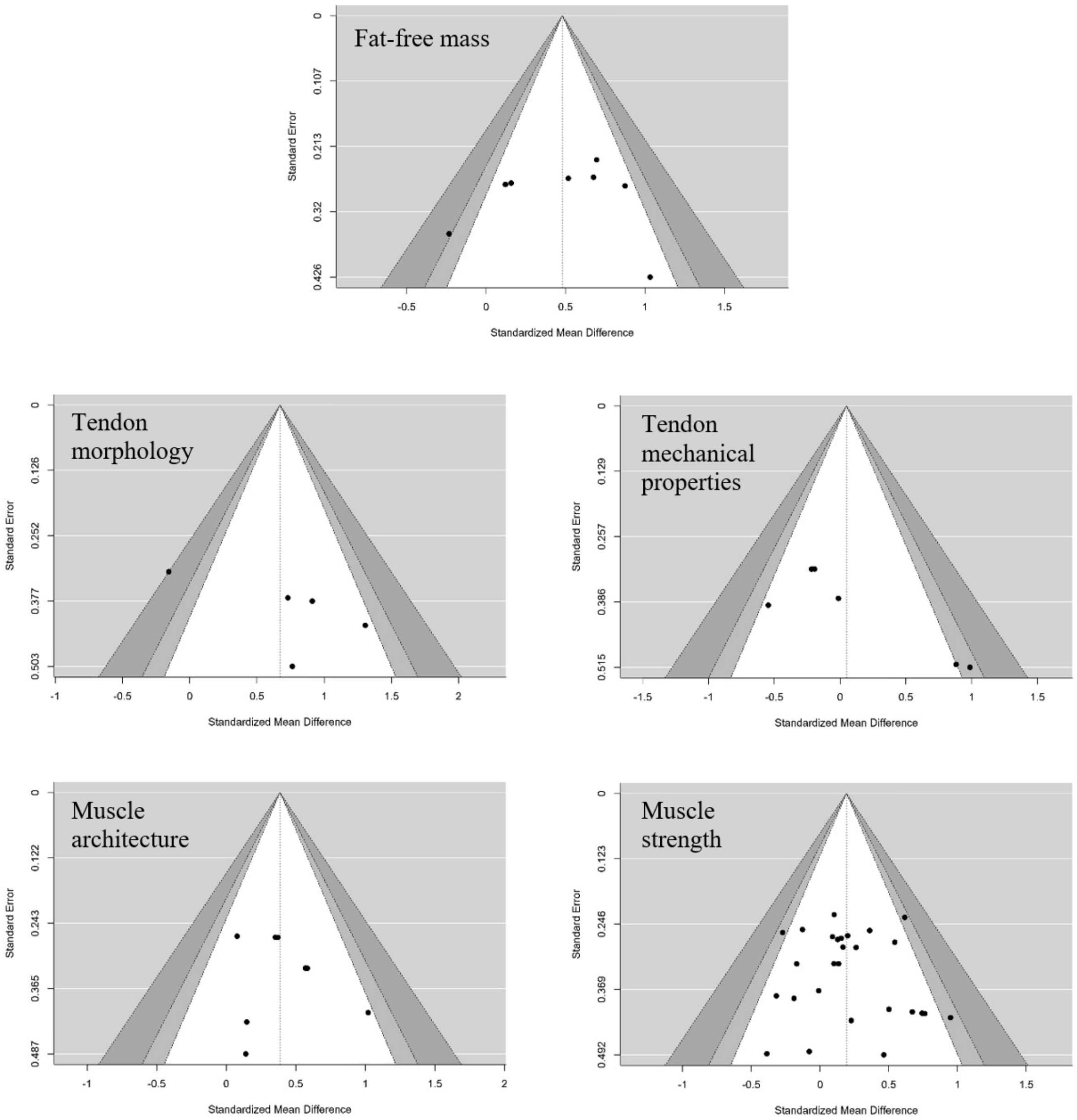

Figure S1: Funnel plots of fat-free mass, tendon morphology, mechanical properties, muscle architecture and strength from top left to bottom right. Black points indicate individual studies.

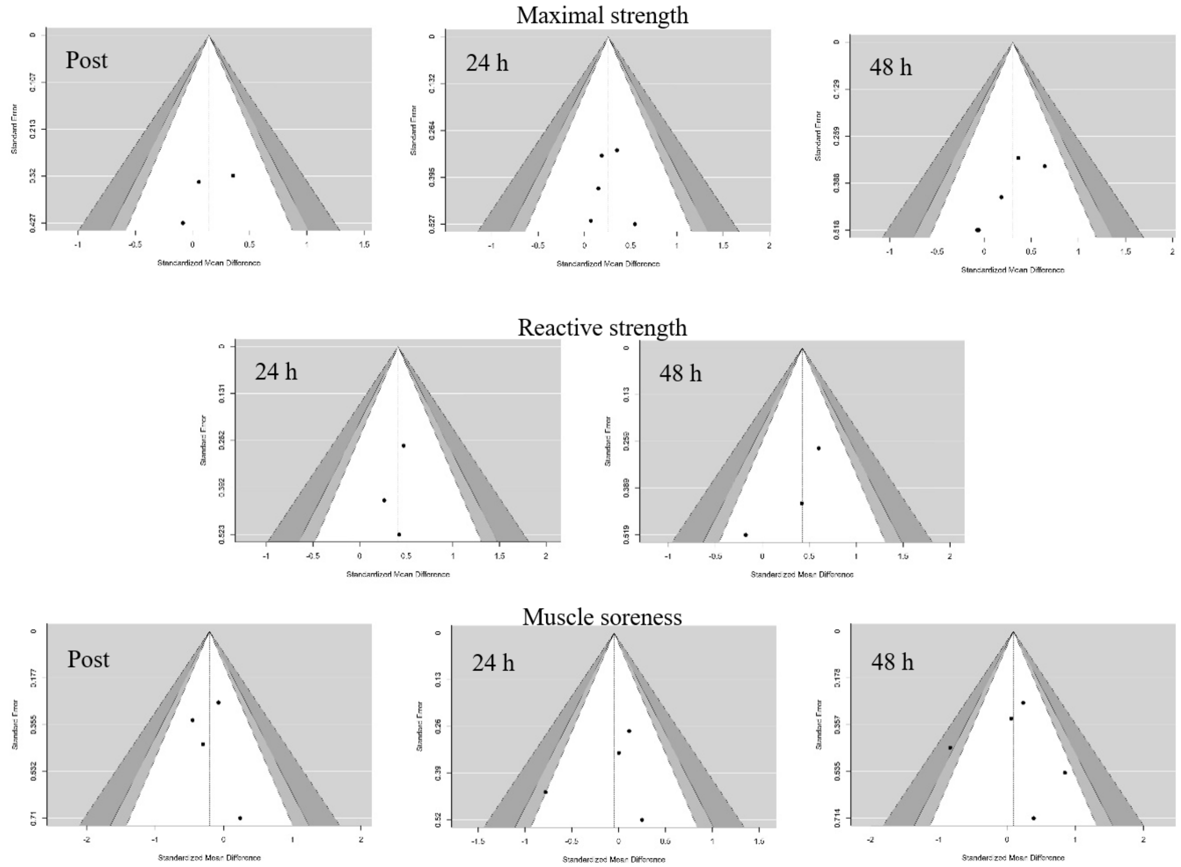

Figure S2: Funnel plots of recovery related parameters. Black points indicate individual studies.
